# One-step biosynthesis of α-ketoisocaproate from l-leucine by an *Escherichia coli* whole-cell biocatalyst expressing an l-amino acid deaminase from *Proteus vulgaris*

**DOI:** 10.1038/srep12614

**Published:** 2015-07-28

**Authors:** Yang Song, Jianghua Li, Hyun-dong Shin, Guocheng Du, Long Liu, Jian Chen

**Affiliations:** 1 Key Laboratory of Carbohydrate Chemistry and Biotechnology, Ministry of Education, Jiangnan University, Wuxi 214122, China; 2 Key Laboratory of Industrial Biotechnology, Ministry of Education, Jiangnan University, Wuxi 214122, China; 3 Synergetic Innovation Center Of Food Safety and Nutrition, Wuxi 214122, China; 4 School of Chemical and Biomolecular Engineeirng, Georgia Institute of Technology, Atlanta 30332, USA

## Abstract

This work aimed to develop a whole-cell biotransformation process for the production of α-ketoisocaproate from L-leucine. A recombinant *Escherichia coli* strain was constructed by expressing an L-amino acid deaminase from *Proteus vulgaris*. To enhance α-ketoisocaproate production, the reaction conditions were optimized as follows: whole-cell biocatalyst 0.8 g/L, leucine concentration 13.1 g/L, temperature 35 °C, pH 7.5, and reaction time 20 h. Under the above conditions, the α-ketoisocaproate titer reached 12.7 g/L with a leucine conversion rate of 97.8%. In addition, different leucine feeding strategies were examined to increase the α-ketoisocaproate titer. When 13.1 g/L leucine was added at 2-h intervals (from 0 to 22 h, 12 addition times), the α-ketoisocaproate titer reached 69.1 g/L, while the leucine conversion rate decreased to 50.3%. We have developed an effective process for the biotechnological production of α-ketoisocaproate that is more environmentally friendly than the traditional petrochemical synthesis approach.

α-Ketoisocaproate (KIC) is an intermediate metabolic product in leucine synthesis and degradation. KIC, used as a nitrogen-free substitutes for leucine, could serve as an integral part of therapy for chronic kidney disease and hepatitis B virus infection to provide patients with their daily requirement of l-leucine. A low-nitrogen diet slows the rate of renal failure by decreasing the accumulation of urea and other nitrogenous waste derived from dietary protein^1^,^2^,^3^,^4^. KIC could also play multifunctional roles in metabolic regulation and thereby reduce protein catabolism^5^, stimulate protein synthesis^6^,^7^, and promote insulin secretion^8^,^9^. With the application of KIC in the food, feed, and pharmaceutical industries, considerable efforts have been devoted to the mass production of KIC. The method most commonly used to produce KIC is chemical synthesis, including the azlactones method, Grignard reagents with diethyloxamates, epoxidation of diethyl alkylidene malonates, double carbonylation, the Fried-l-Crafts acylation method, and the hydantoin method^10^. All these methods need the expensive catalyst or special starting structure, resulting in high costs in KIC production.

Microbial fermentation and enzymatic transformation are alternative processes for the production of KIC. In a previous study, a recombinant *Corynebacterium glutamicum* strain was constructed through metabolic engineering in order to develop a competitive fermentation process, and the maximal KIC titer reached 9.23 g/L^11^. However, except for the low KIC yield, the auxotroph for branch-chained amino acids due to the deletion of *ilvE* is also an obstacle for industrial production. Vogt *et al.* have construct a plasmid free *C. glutamicum* to generate 6.1 g/L KIC^12^. Nevertheless, the production of KIC by metabolic engineering of *C. glutamicum* is still limited by the growth dependent on the l-isoleucine. The whole-cell bioconversion method gives a bright way to low cost process of KIC production. For example, KIC has been prepared using a *Rhodococcus opacus* DSM 43250 whole-cell transformation system, and the KIC titer reached 1275 mg/L^13^,^14^.

With regard to the production of keto acids from amino acids by whole-cell biotransformation, recent studies have focused on recombinant strains expressing enzymes such as amino acid deaminase, dehydrogenase, and transferases. Aminotransferases are not suitable enzymes in mass production because of the need for another keto acid as the essential amino acceptor^15^,^16^. Amino acid dehydrogenases are NADH/NADPH-dependent enzymes that catalyze the oxidative deamination of amino acids to form α-keto acids. The reaction is not cost-effective because of the need for a coenzyme^17^. Therefore, l-amino acid deaminase (l-AAD, EC 1.4.3.2), which has broad substrate specificity, has been investigated for use as an industrial biocatalyst for the catalysis of oxygen-dependent oxidative deamination^18^. Most eukaryotic and prokaryotic l-amino acid oxidases are secreted, whereas l-amino acid deaminases from *Proteus* species are membrane-bound and directly linked to the respiratory chain^19^,^20^. Enzymes from *Proteus* species such as *Proteus vulgaris*, *Proteus mirabilis*, and *Proteus myxofaciens* perform well in heterologous expression systems^21^. A typical example is the production of α-ketoglutaric acid by expressing l-AAD from *P. mirabilis* in *Escherichia coli* BL21 and *Bacillus subtilis*^20^,^22^, resulting in 21.7 and 55.3 μg·mg protein^−1^ min^−1^, respectively^20^. Additionally, l-AAD from *P. mirabilis* can be used to prepare phenyllactic acid^23^. It is reported that α-keto acids were generated in *E. coli* K12 by overexpressing l-AAD from *P. myxofaciens*, during which d- and l-amino acids can be separated as a result^24^.

In this work, we achieved the one-step production of KIC using an *E. coli* whole-cell biocatalyst, which expressed an l-amino acid deaminase from *P. vulgaris*. First, the whole-cell biocatalyst was prepared by expressing the l-amino acid deaminase in *E. coli*. And then the reaction conditions of bioconversion was optimized. Finally, various l-leucine supply strategies were examined to improve the production of KIC.

## Results

### Expression of l-AAD from *P. vulgaris* in *E. coli*

**Figure 1:**
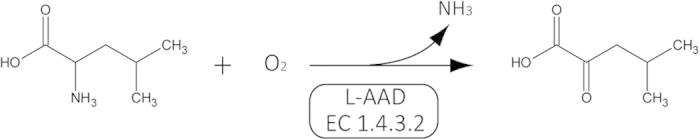
The deamination reaction of l-amino acid deaminase (l-AAD) from *P. vulgaris.*

**Figure 2:**
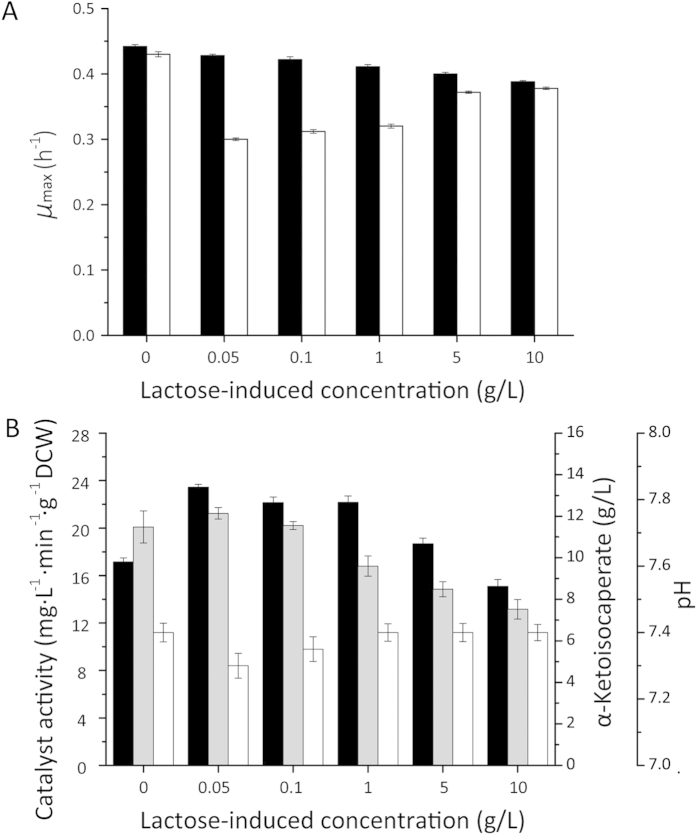
(**A**) Specific growth rate of recombinant strain at different lactose-induction concentration. *E. coli*-pET-28a (+) (black bars), *E. coli*-pET-28a (+)-*lad* (white bars); (**B**) Effect of biocatalyst activity (black bars), α-ketoisocaperate (grey bars) and pH (white bars) at different induction intensities.

The l-AAD from *Proteus* species is a membrane-bound enzyme that is difficult to overexpress in *E. coli*, in which it affects cell growth^25^. Structure prediction using the prediction software TMHMM 2.0(website: http://www.cbs.dtu.dk/services/TMHMM-2.0) has confirmed that the enzyme is a type II membrane protein^26^. l-AADs are flavoenzymes that typically catalyze deamination with the release of hydrogen peroxide, giving the enzymes antibacterial activity and cytotoxic properties^27^,^28^. However, the deamination mechanism of the membrane-bound deaminase is not clear. The l-AAD reaction (Fig. 1) showed the elements in the catalyst process, as well as the changes of chemical structures between α-ketoisocaproate and leucine. To determine whether the expression of l-AAD affected cell growth and the activity of the whole-cell biocatalyst, a set of inducer concentrations was evaluated. As shown in Fig. 2A, the maximum specific growth rate decreased in engineered l-AAD-expressing strains compared with nontransformed cells, indicating that the l -AAD from *P. vulgaris* had negative effect on the overexpression strains. The expression of l-AAD resulted in sufficient amino acid deamination, which disturbs the growth of host^22^. The increase of intracellular concentration of oxidized corresponding amino acids changed the redox status in *E. coli*^22^. Moreover, the biocatalyst activity varied according to the lactose concentration. A lactose induction concentration of 0.05 g/L resulted in the highest whole-cell biocatalyst activity and KIC production, as well as a good cell growth rate. Increasing the inducer concentration decreased the biocatalyst activity and KIC production.

### Optimization of KIC production by the whole-cell biocatalyst

**Figure 3:**
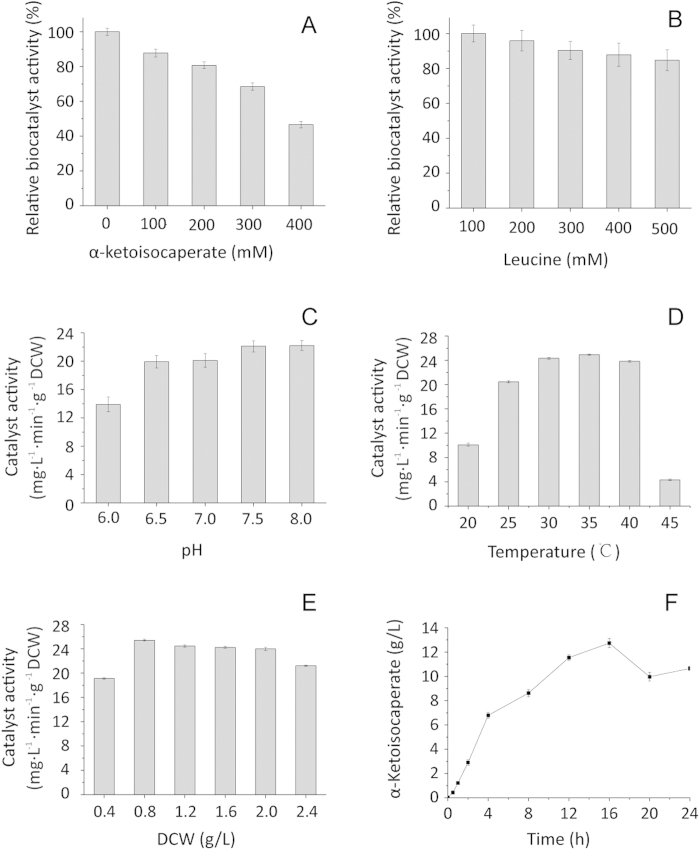
The effect of KIC, substrate, pH, temperature, DCW, and time on whole-cell biotransformation. (**A**) Influence of KIC concentration; (**B**) effect of substrate concentration; (**C**) Influence of pH; (**D**) Influence of temperature; (**E**) Influence of biocatalyst; (**F**) KIC production at 0.8 g/L DCW over time.

In order to use the enzyme for KIC production, the enzyme properties were evaluated. We analyzed l-AAD activity in the presence of different KIC concentrations (0 g/L–52.05 g/L) and leucine concentrations (0 g/L–65.59 g/L). Product inhibition of l-AAD was assessed by measuring KIC production. The biocatalyst activity decreased sharply as KIC concentration increased (Fig. 3A). Substrate inhibition was not apparent because of the insolubility of supersaturated l-leucine. (Fig. 3B). The whole-cell biocatalyst activity under different pH conditions was analyzed in KH_2_PO_4_-K_2_HPO_4_ buffers (Fig. 3C). The highest biocatalyst activity of 25.11 mg·L^−1^ min^−1^·g^−1^ DCW was obtained at pH 7.5, consistent with a previous study, which found that the optimal pH for a similar *Proteus* enzyme was in the alkaline range 22. Fig. 3D shows the effect of reaction temperatures ranging from 20 to 45 °C. The highest biocatalyst activity was 24.92 mg·L^−1^ min^−1^·g^−1^ DCW at 35 °C. The biocatalyst activity remained almost constant from 30 to 40 °C, but decreased sharply above 40 °C and below 30 °C. The effect of the biocatalyst concentration on biocatalyst activity was then evaluated. The highest biocatalyst activity was 24.01 mg·L^−1^ min^−1^·g^−1^ DCW at 0.8 g/L cells, and activity decreased at higher cell concentrations (Fig. 3E). This indicated that a high cell density was harmful to the reaction. To investigate KIC production at different times with the optimal cell density, we monitored KIC production over a 24-h period. As shown in Fig. 3F, bioconversion was evaluated in a shake flask using 0.8 g/L cells and 13.1 g/L leucine. KIC production reached 12.7 ± 0.38 g/L at 16 h and then decreased at later time points. With regard to the conversion efficiency and economical industrial application, 16 h was the ideal operation time. Overall, the optimal conditions were 0.8 g/L cells at 35 °C for 16 h. The conversion rate of l-leucine was 97.8% under the optimal conditions with 13.1 g/L leucine.

### Effect of l-leucine transporters on whole-cell biocatalyst activity

**Figure 4:**
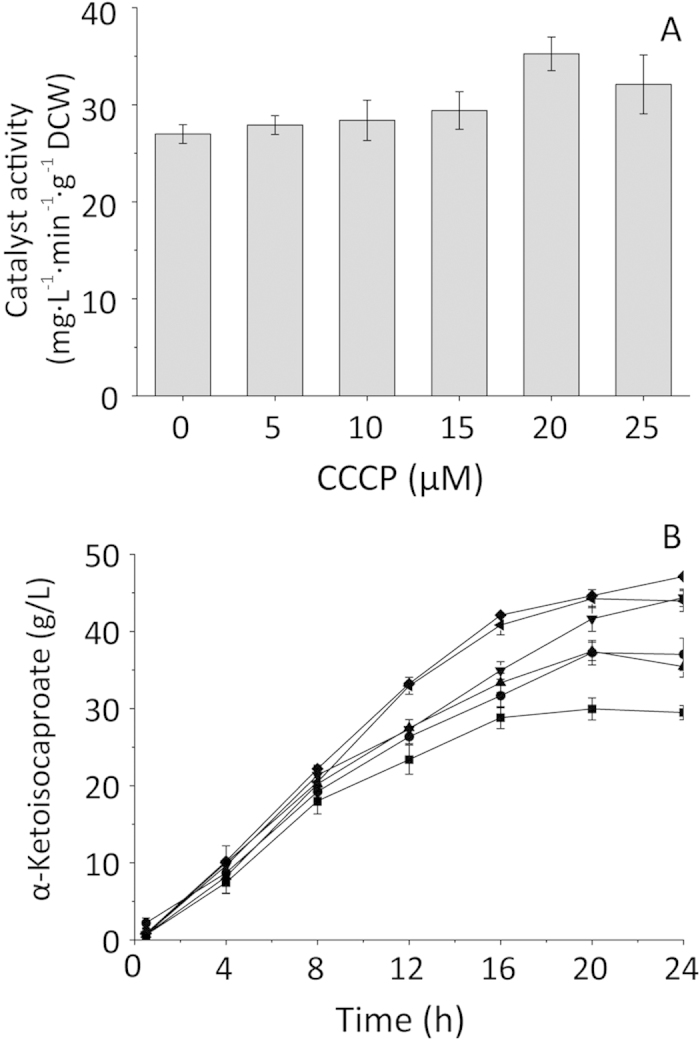
The comparison of biocatalyst activity by whole-cell biocatalyst and production at different concentration of CCCP. (**A**) biocatalyst activity at different CCCP concentration; (**B**) α-ketoisocaporate accumulation during bioconversion in 52.47 g/L substrate. CCCP concentration: 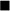: 0 μM, 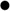: 5 μM, 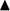: 10 μM, 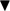: 15 μM, 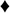: 20 μM, 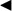: 25 μM.

The uptake of l-leucine depends on two different transport systems. The high-affinity branched-chained amino acid transport systems LivFGHMJ and LivFGHMK comprise members of the ATP binding cassette (ABC) transporter family. livJ transports three amino acids, while livK only transports l-leucine. The low-affinity LIVCS transporter BrnQ requires an inwardly directed Na^+^ gradient (ΔpNa)^29^. Therefore, we used protonophores to uncouple the agents that catalyze electrogenic proton movement, in order to reduce ATP generation and inhibit livK, livJ, and BrnQ transport efficiency. The biocatalyst activity was tested by adding carbonyl cyanide-3-chlorophenylhydrazone (CCCP) to the reaction mixture at different concentrations. The results showed that CCCP increased the biocatalyst activity at concentrations below 20 mM (Fig. 4). These amounts of CCCP inhibited the uptake of l-leucine and KIC. However, for industrial purposes, knockout of *liv*K and *Brn*Q to limit leucine transport is recommended.

### Effect of different l-leucine supply strategies on KIC production

**Figure 5:**
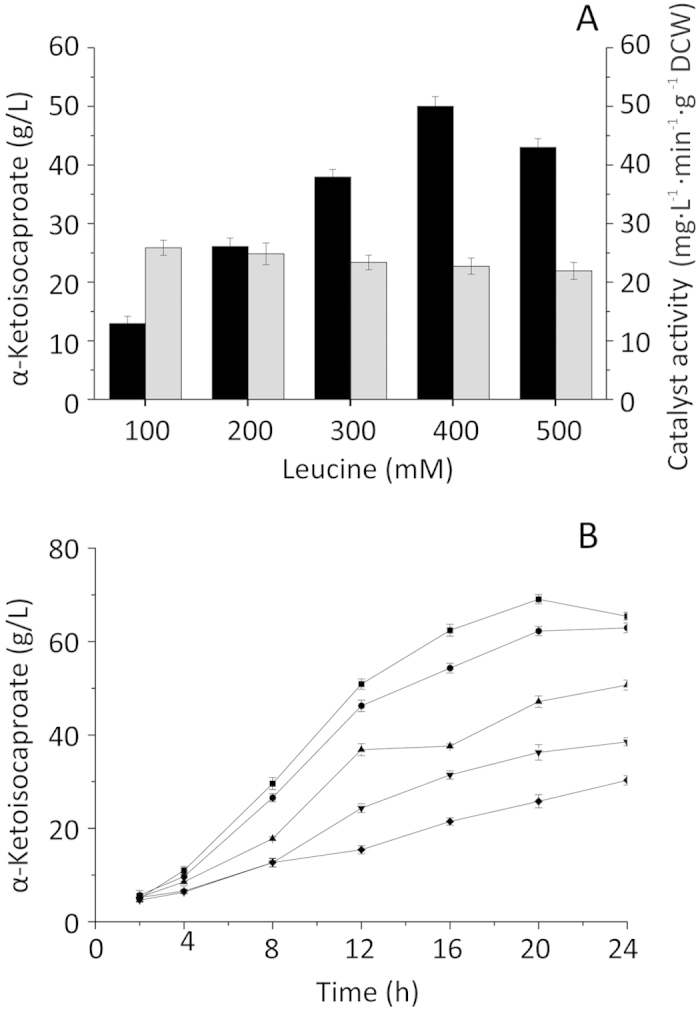
The effect of different feeding strategies on production of α-ketoisocaperate and biocatalyst activity. (**A**) The production of α-ketoisocaperate (black bars) and biocatalyst activity (grey bars) with different initial substrate concentrations; (**B**) The α-ketoisocaperateproductionat different feeding interval time. 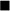: 2 h, 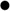: 4 h, 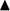: 6 h, 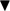: 8 h; 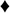: 10 h.

Because of the poor solubility of l-leucine, only two supply strategies could be used in KIC production. Neither constant-rate supply nor exponential supply could be used. Therefore, the effects of batch and interval leucine feeding on KIC production were assessed in a flask reactor. As shown in Fig. 5A, during batch bioconversion, leucine substrate concentrations of 13.1 g/L to 65.5 g/L were investigated. Because l-leucine is an aliphatic amino acid with poor water solubility, the reaction was carried out in leucine solution with powder floating on the surface of the liquid. The leucine powder dissolved as l-leucine was used. KIC production reached 50.0 g/L with a leucine conversion rate of 96.1%, suggesting that a low concentration of leucine (13.1 g/L, 26.2 g/L, 39.3 g/L) does not provide enough substrate for KIC synthesis, whereas a higher concentration of leucine (65.5 g/L) reduces the mixing transfer efficiency and oxygen transfer rate. The biocatalyst activity decreased as the leucine concentration increased, and the highest biocatalyst activity was obtained when the leucine concentration was 13.1 g/L. This indicated that a high substrate concentration inhibited enzyme activity.

To overcome inhibition due to a high substrate concentration, we added leucine at regular intervals and investigated the effect on KIC production. Compared with the batch bioconversion method, leucine addition at intervals resulted in higher KIC production because of the high biocatalyst activity. Figure 5B shows the temporal profile of KIC production during the biotransformation. Feeding leucine at a high rate appeared to increase the KIC yield and shorten the time needed for the KIC yield to peak. The KIC titer reached 69.1 g/L when leucine was added at 2-h intervals (from 0 to 22 h), while the leucine bioconversion rate decreased to 50.3%. The interval leucine supply method produced more KIC than did the batch bioconversion method (69.06 g/L to 50.02 g/L) (Table 1). The interval supply method might favor higher biocatalyst activity with less mass interference. Nevertheless, the bioconversion rate was lower than that observed with the batch bioconversion method, which affected purification. Thus, the batch method is more effective for the production of KIC.

### Application of recombinant *E. coli* for immobilization

**Figure 6:**
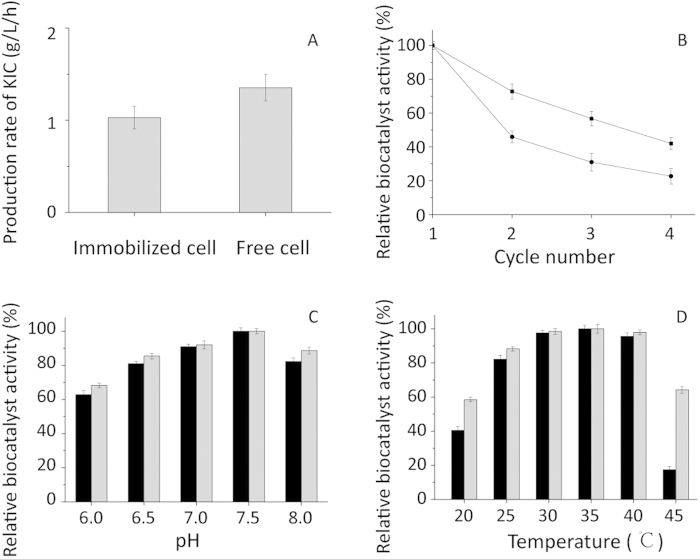
Immobilization and recyclablilty of the whole-cell biocatalyst. (**A**) Comparision of immobilization on whole-cell biocatalyst activity. (**B**) The comparision of the recycle ability between the free cells and immoblized cells. [straight line with square mark (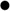) for free cell and dot line with triangle mark (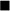) for immobilized cell]; Comparison of the pH (**C**), and thermal (**D**) (black bar for free and light grey bar for immobilized recombinant whole cell biocatalyst).

For industrial production, immobilization is a desirable way to achieve continuous operation without cell washout. We analyzed how immobilization affected the long-term bioconversion efficiency. The initial reaction rate with immobilized cells was 75.99% of the reaction rate with free cells when the same amounts of cells were used (Fig. 6A). The decrease in biocatalyst activity was due to shielding by alginate, which decreased the contact area between the cells and the substrate. However, the immobilized cells were better when the conversion time was long. The reusability increased 19.24% when compared with that of free cells (Fig. 6B). In addition, the immobilized cells had greater pH and thermal stability (Fig. 6C,D). Because l-AAD is a membrane-bound enzyme, l-AAD is exposed to the reaction solution. Consequently, l-AAD is stabilized by the networks in the alginate beads, which protect the enzyme from the effects of the H^+^ ions. In addition, immobilization is an effective way to separate product and cells. Moreover, the technique could solve the problem of product inhibition, resulting in an effective production process.

## Discussion

In this study, l-AAD was expressed in *E. coli* BL21 (DE3) cells. The conversion rate of l-leucine was 97.8% under optimal conditions with 13.1 g/L leucine. With different supply strategies, a higher KIC production (69.1 g/L) was obtained at 20 h when leucine was added at intervals of 2 h. Compared with chemical synthesis, bioconversion is more cost effective with regard to the use of leucine as a starting material.

In previous studies, the highest production of KIC was 9.23 g/L achieved by metabolically engineering *C. glutamicum* at 25 h with glucose as the substrate^11^. However, two kinds of branch-chained amino acids were required to maintain the growth of the strain due to the amino acid auxotrophy, and acetate (1%, w/v) needed to be added to the broth at suitable times to stimulate the synthesis of acetyl-CoA. Modified *C. glutamicum* strains have been constructed by changing the codons in *ilvE* and expressing endogenous genes. These changes resulted in the production of 6.1 g/L KIC in the presence of 0.13 g/L l-isoleucine^12^. Low production and amino acid auxotrophy limit the application of fermentation in industrial processes. However, whole-cell biotransformation methods solve the problem of low KIC production and produce KIC at an efficient rate.

Compared with other l-AADs and l-amino acid oxidases (LAAOs), l-AAD from *P. vulgaris* produces KIC more efficiently. The deaminases from *Proteus* species require no cofactors. Furthermore, the l-AAD enzymes from *Proteus* species do not use the usual oxidative deamination reaction because no hydrogen peroxide is generated during the deamination^27^,^28^. A typical oxidative deamination reaction can be divided into two main half-reactions. First, the amino acid is oxidized into the α-keto acid and ammonium with the release of a hydride equivalent. Second, the hydride is usually transferred to O_2_ to generate hydrogen peroxide. Thus, oxygen is required in the deamination reaction^27^. A detailed mechanism for l-AADs is not available because crystal structures of membrane proteins are difficult to obtain. Furthermore, l-AAD from *P. vulgaris* has the advantage of heterologous expression compared with l-AAO, because no hydrogen peroxide is generated. The first heterologous system for the expression of l-AAO was developed using *Streptomyces lividans* as the host. l-AAO from *Rhodococcus opacus* DSM 43250 was expressed in an active form and resulted a protein expression of 0.2 mg/L, while expression in *E. coli* resulted in the insoluble protein^30^. The expression of l-AAO with broad substrate specificity has been attempted in various expression hosts^31^. However, a common observation is that the level of l-AAO expression is very low. l-AAD from *Proteus* has the similar broad substrate specificity with l-AAO, while could be easily expressed in *E. coli* and other common hosts. l-AAD from *P. mirabilis, P. vulgaris* have been expressed in *E. coli* BL21 and *Bacillus subtilis*^22^,^32^. The l-AAD from *P. vulgaris* has higher catalytic activity on the hydrophobic amino acids, which is more suitable for leucine bioconversion. Above all, l-AAD from *P. vulgaris* is an ideal choice for KIC production.

To improve KIC production, enzyme activity is the key factor in bioconversion. Therefore, we studied enzyme inhibition by l-leucine and KIC. Enzyme activity in high KIC concentrations changed considerably, which suggested that product inhibition was a critical and limiting factor in KIC production. However, the addition of substrate did not affect enzyme activity, probably because leucine was not soluble enough to inhibit activity. Product inhibition explained why the highest bioconversion rate occurred at low product concentrations (97%, 13.1 g/L leucine). Limiting leucine transport also improved the KIC conversion rate. CCCP blocked the energy needed for leucine transport and glycolytic pathways, thereby increasing the biocatalyst activity.

The environmental friendly process for α-ketoisocaproate production through biotransformation is more attractive for the industrial production of KIC. However, product inhibition limited KIC production. Therefore, to improve production, development of a feedback-resistant l-AAD is desirable.

## Methods

### Microorganisms and media

The strain was constructed by cloning and expressing the l-AAD gene in *E. coli* BL21 (DE3). The oligonucleotides, strain, plasmids used in this article are listed in Table 2. The l-AAD gene was amplified from the *P. vulgaris* genome and inserted between the *Bam*HI and *Xho*I restriction sites in the pET-28a plasmid. The recombined plasmid was then transformed into *E. coli* BL21 (DE3). The construct was confirmed by gene sequencing.

The EZ-10 Spin Column Plasmid DNA MiniPreps kit, DNA purification kit, restriction enzymes, and T4 DNA ligase were purchased from Takara (Dalian, China). The standard sample of KIC was obtained from Sigma-Aldrich (St. Louis, MO, USA). Ampicillin was acquired from Amresco (Solon, OH, USA). CCCP was purchased from Sigma-Aldrich (St. Louis, MO, USA). *E. coli* seed cultures were initiated in Luria-Bertani (LB) medium. l-AAD was produced and whole-cell biocatalysts were grown in Terrific Broth (TB) with the same antibiotic.

### Preparation of the whole-cell biocatalyst and measurement of bacterial growth under different induction conditions

The recombinant strain was inoculated in 25 mL of LB medium supplemented with kanamycin at a final concentration of 40 μg/mL and cultivated at 37 °C overnight. Thereafter, 2% of the seed culture was added to the fermentation medium (TB) with 40 μg/mL kanamycin and cultivated at 37 °C and 200 rpm. When the OD_600_ reached 0.6, different concentrations of lactose were immediately added to the broth to final concentrations of 0 g/L, 0.05 g/L, 0.1 g/L, 1 g/L, 5 g/L, and 10 g/L. Growth curves were measured every hour until the stationary phase as shown by measuring the OD_600_. The measurement was performed in three replicates. Growth characteristics were fitted using OriginPro 8.5 and the maximum specific growth rate (μ_max_) was recorded.

Cells were harvested at the end of the exponential phase of growth by centrifugation at 10, 000 × *g* at 4 °C for 5 min. The cells were washed twice with sterilized water and resuspended in the leucine solution with CCCP used for transformation. The reaction stopped by centrifugation at 8000 × *g* for 10 min, and the supernatant was recovered for the measurement of KIC with high-performance liquid chromatography (HPLC), as described below.

### Cell density and biocatalytic activity assays

Aliquots (1 mL) were drawn from the flask and diluted to the appropriate concentration. The OD_600_ was determined using a UVmini-1240 spectrophotometer (Shimadzu, Kyoto, Japan). The dry cell weight (DCW) was calculated from the OD_600_ according to the following Eq. (1):
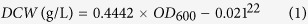


To determine the biocatalyst activity, washed cells were dissolved in 1 mL of leucine solution (13.1 g/L, pH 7.5, CCCP 20 μM) and suspended gently. The reaction was performed in 5-mL centrifugation tubes at 35 °C and 200 rpm for 30 min. The reaction was stopped by centrifugation at 10,000 × *g* at 4 °C for 5 min. The supernatant was recovered for the measurement of KIC using HPLC, as described below. The biocatalyst activity was calculated according to the following Eq. (2):
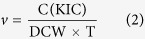
where *ν* is the catalyst activity (the amount of KIC formed by 1 g of cells per min, mg·L^−1^ min^−1^·g^−1^ DCW), *C*_(KIC)_ is the concentration of KIC (g/L), DCW is the dry cell weight (g/L), and the reaction time *T* was 30 min. The biocatalyst activity could reflect reaction rate as described in supplementary materials.

### Optimization of substrate concentration, product concentration, pH, temperature, cell density, substrate concentration, induction time, and CCCP concentration

For the optimization of all variables, the reaction was performed in a 5-mL centrifugation tube for 30 min. The biocatalyst activity was tested as described above. To optimize the effect of substrate concentration, cells (1 g/L ) were incubated at 37 °C in different leucine concentrations. The effect of product concentration was tested in 13.1 g/L leucine solution with 1 g/L cells at 37 °C. For pH optimization, the reaction was performed with 1 g/L cells at 37 °C in KH_2_PO_4_-K_2_HPO_4_ buffers (pH 6.0–8.5). For temperature optimization, the reaction included 1 g/L cells and l-leucine solution (13.1 g/L, pH 7.5), with the temperature ranging from 20 to 45 °C. DCW was optimized using 13.1 g/L l-leucine at pH 7.5 and 35 °C. For KIC production with 0.8 g/L cells, the reactions were performed in a 500-mL shake flask in 50 mL of reaction solution with CCCP. Samples (1 mL) were taken from the reaction container and centrifuged at 8000 × *g* for 10 min. The supernatant was tested with HPLC.

### l-leucine supply strategies

The bioconversions were performed in a 500-mL shake flask in 50 mL of reaction solution. The batch bioconversion method used 0.8 g/L cells mixed with different initial concentrations of leucine (13.1 g/L, 26.2 g/L, 39.3 g/L, 52.4 g/L, or 65.5 g/L) and 20 μM CCCP. Because leucine is an aliphatic amino acid with poor solubility in an aqueous solution, the leucine powder affects the activity of the mass transfer efficiency. To maximize the enzyme activity, the interval supply approach was tested as follows. The initial l-leucine concentration was 13.1 g/L with 20 μM CCCP, and 13.1 g/L l-leucine powder was added once at different times (2 h, 4 h, 6 h, 8 h). A sample was collected every 4 h. KIC production and biocatalyst activity were tested according to the methods described above.

### Cell immobilization

Cells were harvested during the exponential growth phase and mixed with sodium alginate solution (3%, w/v). The cell density was 0.8 g/L. The mixture was passed through a peristaltic pump into a CaCl_2_ solution (136 mM) and then incubated for 1 h at 4 °C. The beads were harvested by filtration and washed twice with sterile distilled water.

Recyclability was tested in repeated-batch conversion experiments. The beads were obtained by filtration, and the same amount of leucine solution was added to initiate the deamination reaction. Free cells were collected by centrifugation and redissolved in leucine solution. The beads and the free cells were washed twice before the reaction. The biocatalyst activity was assessed by measuring the KIC concentration for 30 min in KH_2_PO_4_-K_2_HPO_4_ buffers (pH 6.0–8.5) at 20–45 °C. Enzyme recycling was performed once for 24 h and repeated 4 times. The highest whole-cell biocatalyst activity was defined as 100%.

### Analysis of keto acid concentrations using HPLC

The KIC concentration in the reaction mixture was determined using HPLC (Agilent 1200 series, Santa Clara, CA, USA) with an Agilent ZORBAX SB-Aq column (4.6 × 250 mm, 5 μm). The mobile phase was a mixture of 90% diammonium phosphate (pH 2.50) and 10% methanol with a flow rate of 0.8 mL/min. The column temperature was maintained at 35 °C, and the injection volume was 10 μL. KIC was detected at a wavelength of 203 nm with a UV detector.

### Statistical analysis

All experiments were performed at least three times, and the results were expressed as the mean ± standard deviation (n = 3). Data were analyzed using Student’s t-test.

## Additional Information

**How to cite this article**: Song, Y. *et al.* One-step biosynthesis of α-ketoisocaproate from L-leucine by an *Escherichia coli* whole-cell biocatalyst expressing an L-amino acid deaminase from *Proteus vulgaris. Sci. Rep.* **5**, 12614; doi: 10.1038/srep12614 (2015).

## Supplementary Material

Supplementary Information

## Figures and Tables

**Table 1:** Comparison of α-ketoisocaperate production with different leucine supply approaches.

	**batch bioconversion method**					**Interval leucine supply method**				
	13.1 g/L	26.2 g/L	39.3 g/L	52.4 g/L	65.5 g/L	2 h	4 h	6 h	8 h	10 h
Maximum KIC concentration (g/L)	12.96 ± 1.22	26.07 ± 1.47	37.95 ± 1.29	50.02 ± 1.64	43.02 ± 1.48	69.06 ± 1.00	62.92 ± 1.05	50.69 ± 1.08	38.52 ± 0.85	30.27 ± 1.00
Bioconversion rate (mol/mol)	0.99 ± 0.01	1.00 ± 0.03	0.97 ± 0.002	0.96 ± 0.04	0.66 ± 0.03	0.50 ± 0.03	0.80 ± 0.02	0.97 ± 0.01	0.98 ± 0.02	0.77 ± 0.02
KIC productivity (g/L/h)	0.65 ± 0.06	1.30 ± 0.07	1.89 ± 0.06	2.51 ± 0.08	2.51 ± 0.07	3.27 ± 0.05	2.62 ± 0.04	2.11 ± 0.04	1.60 ± 0.03	1.26 ± 0.04
Yield of KIC on cell (*Y*_*KIC/X*_)	0.81 ± 0.07	1.62 ± 0.09	2.37 ± 0.08	3.12 ± 0.10	2.68 ± 0.09	4.31 ± 0.06	3.40 ± 0.05	3.16 ± 0.05	3.11 ± 0.04	2.46 ± 0.05

**Table 2:** Oligonuleotide primers, plasmids and strains used in this study.

**Pimers/Plasmids/Strains**	**Nucleotide sequence (5′-3′)/description**	**Restriction enzyme/Sources**
Primers		
L-AAD_F	CGCGGATCCATGGCGATATCTAGAAGAAAATTTA	*Bam*HI
L-AAD_R	CCGCTCGAGTTAGAATCTGTAAAGACTAAATGGTTT	*Xho*I
Plasmids		
pET28a		Invitrogen, Carlsbad, CA
Strains		
*E. coli* BL21 (DE3)		Invitrogen, Carlsbad, CA
*P. vulgaris*		Japan Collection of Microorganism Saitama, Japan
