# Supplementary material for: One-step biosynthesis of α-ketoisocaproate from l-leucine by an Escherichia coli whole-cell biocatalyst expressing an l-amino acid deaminase from Proteus vulgaris
Source: Sci Rep. 2015 Jul 28;5:12614. doi: 10.1038/srep12614 (PMC4517468; doi:10.1038/srep12614)
Supplement: Supplementary Information [file srep12614-s1.docx]

SUPPLEMENTAL INFORMATION

**One-step biosynthesis of α-ketoisocaproate from l-leucine by an *Escherichia coli* whole-cell biocatalyst expressing an l-amino acid deaminase from *Proteus vulgaris***

Yang Song^1,2,3^, Jianghua Li ^1,2,3^, Hyun-dong Shin^4^, Guocheng Du^1,2,3*^, Long Liu^1,2,3*^, Jian Chen^2,3^

^1^Key Laboratory of Carbohydrate Chemistry and Biotechnology, Ministry of Education, Jiangnan University, Wuxi 214122, China

^2^Key Laboratory of Industrial Biotechnology, Ministry of Education, Jiangnan University, Wuxi 214122, China

^3^Synergetic Innovation Center Of Food Safety and Nutrition, Wuxi 214122, China

^4^School of Chemical and Biomolecular Engineeirng, Georgia Institute of Technology, Atlanta 30332, USA

***Corresponding authors:**

Guocheng Du, Tel.: +86-510-85918309, Fax: +86-510-85918309, E-mail: [gcdu@jiangnan.edu.cn](mailto:gcdu@jiangnan.edu.cn);

Long Liu, Tel.: +86-510-85918312, Fax: +86-510-85918312, E-mail: [longliu@jiangnan.edu.cn](mailto:longliu@jiangnan.edu.cn).

SUPPLEMENTARY FIGURE LEGENDS

Supplemental Figure S1. Comparison of catalyst activity and reaction rate. The slope is reaction rate, and the ratio of third point is catalyst activity.

SUPPLEMENTARY FIGURES

(Fig.S1)
